# Insights Into Remote Ischemic Conditioning miRNA Effects on Brain Endothelial Cells During Ischemia–Reperfusion

**DOI:** 10.1111/micc.70060

**Published:** 2026-05-01

**Authors:** Katrine Tang Stenz, Jesper Just, Zenghui Huang, Thomas Ravn Lassen, Kristian Vissing, Xiu‐Jie Wang, Kim Ryun Drasbek

**Affiliations:** ^1^ Department of Clinical Medicine, Center of Functionally Integrative Neuroscience Aarhus University Aarhus Denmark; ^2^ Sino‐Danish College (SDC) University of Chinese Academy Sciences Beijing China; ^3^ Department of Molecular Medicine Aarhus University Hospital Aarhus Denmark; ^4^ Institute of Genetics and Developmental Biology Chinese Academy of Sciences Beijing China; ^5^ Department of Cardiology Aarhus University Hospital Aarhus Denmark; ^6^ Department of Public Health Aarhus University Aarhus Denmark; ^7^ School of Future Technology University of Chinese Academy of Sciences Beijing China

**Keywords:** human brain microvascular endothelial cells, miRNA, oxygen and glucose deprivation, remote ischemic conditioning, RNA‐seq, stroke

## Abstract

**Objective:**

Acute ischemic stroke (AIS) is one of the leading causes of death and disabilities, and as such, it is of utmost importance to identify novel treatment options. Remote ischemic conditioning (RIC) is a promising non‐invasive treatment that is thought to activate the body's own protective mechanisms against damaging ischemia. Here, we study the transcriptomic impact of microRNAs (miRNAs) that are upregulated by RIC.

**Methods:**

Using RNA sequencing, we investigated the transcriptional changes in human brain microvascular endothelial cells (HBMECs) transfected with four selected RIC‐upregulated miRNAs (RIC‐miRNAs), miR‐16‐5p, miR‐144‐3p, miR‐182‐5p, and miR‐451a, under oxygen and glucose deprivation (OGD) and reoxygenation—mimicking the initial stages of AIS.

**Results:**

Pronounced transcriptional changes were present after RIC‐miRNA transfection, with 149 unique downregulated and 212 upregulated differentially expressed genes in HBMECs after OGD and RIC‐miRNA transfection compared to all other conditions. These genes were involved in pathways of energy metabolism and cell cycle regulation.

**Conclusion:**

Our study suggests that the selected RIC‐miRNAs regulate pathways that may facilitate endothelial cell survival, recovery, and remodeling events from ischemic damage, adding to the knowledge of the pathways affected by RIC during stroke.

**Abbreviations**
AIS: acute ischemic stroke
BFRRE: blood flow restricted resistance exercise
DEGs: differentially expressed genes
EVs: extracellular vesicles
EVT: endovascular thrombectomy
HBMECs: human brain microvascular endothelial cells
IRI: ischemia–reperfusion injury
miRNA: microRNA
OGD: oxygen and glucose deprivation
RIC: remote Ischemic conditioning
tPA: tissue plasminogen activator

## 1 Introduction

Stroke is one of the leading causes of death and disabilities worldwide [1]. Acute ischemic stroke (AIS) is the most common type of stroke in which cerebral arteries are occluded, leading to oxygen and nutrient depletion ultimately leading to blood brain barrier breakdown [2]. During an ischemic stroke, the delivery of glucose is impaired, which leads to rapid ATP depletion. This in turn disrupts the membrane potential and leads to energy failure due to calcium influx and excitotoxicity [3]. Consequently, many cells undergo apoptosis and for those that do not, cell cycle arrest is induced, and non‐essential pathways are inhibited to preserve energy [4]—a mechanism of protection.

Today, only two acute reperfusion treatments are available for AIS: intravenous thrombolysis by tissue plasminogen activator (tPA) and endovascular thrombectomy (EVT), which have resulted in large reductions in mortality and disabilities after AIS [5, 6, 7]. However, both treatments have narrow treatment windows of 4.5–6 h after symptom onset [6, 8, 9, 10]. Today, only 1%–8% of AIS patients in developed countries receive reperfusion therapies due to late arrival at the hospital and in‐hospital diagnostic delays [11, 12]. In the ischemic phase, millions of brain cells die every minute [13], and even after reperfusion, ischemia–reperfusion injury (IRI) can accelerate the damage and ultimately lead to edema or hemorrhagic transformation [14]. Thus, there is an urgent need for neuroprotective strategies and for add‐on treatments to expand the treatment window. One promising neuroprotective strategy is remote ischemic conditioning (RIC), which has been shown to reduce infarcts and improve neurological function in experimental stroke models (reviewed by [15]). As RIC is performed by repeated cycles of transient (i.e., few minutes) complete arterial blood flow occlusion in an arm or leg, followed by reperfusion, it is easily translated to clinical use and can be initiated in the ambulance. In clinical studies, RIC has been linked to several beneficial effects, including stroke protection by either avoiding stroke recurrence after twice daily repeated RIC (180 to 300 days) [16, 17], decreased stroke scores [18, 19, 20], reduced inflammation [17], increased neuroprotection [19], increased tissue survival [21], improved perfusion after stroke [16], and decreased total lesion volume following carotid artery stenting surgery [22].

We and others have previously shown that the cytoprotective effect of ischemic interventions like RIC can be attributed to the nano‐sized extracellular vesicles (EVs) released into the blood. Specifically, murine post‐RIC EVs reduced infarct size 24 h post stroke in a murine stroke model [23], while post‐RIC EVs from healthy male volunteers attenuated IRI in primary human brain microvascular endothelial cells (HBMECs) [24] as well as decreased the infarct size in the rat Langendorff myocardial infarction model [25]. EVs are thought to be important for intercellular communication and are packaged with DNA, RNA, and proteins. Of special interest is microRNA (miRNA), which can alter gene expression in remote target cells by repressing the translation of specific mRNAs [26] and are reported to change after RIC in healthy volunteers and stroke patients [27]. However, little consensus of miRNA changes is found among the human studies, which are quite diverse regarding patient cohorts, RIC protocols, and miRNA analysis techniques. Previously, we found miR‐16‐5p, miR‐144‐3p, and miR‐451a to be upregulated in post‐RIC EVs [25]. Moreover, using a RIC related exercise paradigm, blood flow restricted resistance exercise (BFRRE, characterized by partial arterial and complete venous occlusion to an exercising limb), we found miR‐182‐5p together with miR‐451a and miR‐16‐5p to be upregulated in post‐BFRRE EVs [28]. Noteworthy, miR‐182‐5p has also been shown to attenuate cerebral IRI in a stroke rat model [29]. We hypothesize that these RIC increased EV‐associated miRNAs affect specific mechanisms for tissue protection. To investigate the protective cell reprogramming potential of these four miRNAs during AIS, we studied the transcriptomic changes after oxygen–glucose deprivation and reperfusion (OGD, an in vitro stroke model), which points to biological pathways responsible for protection against damaging ischemia in HBMECs. Our results could form the basis for studies evaluating molecular RIC protective targets for stroke treatments.

## 2 Materials and Methods

### 2.1 Experimental Design

**FIGURE 1:**
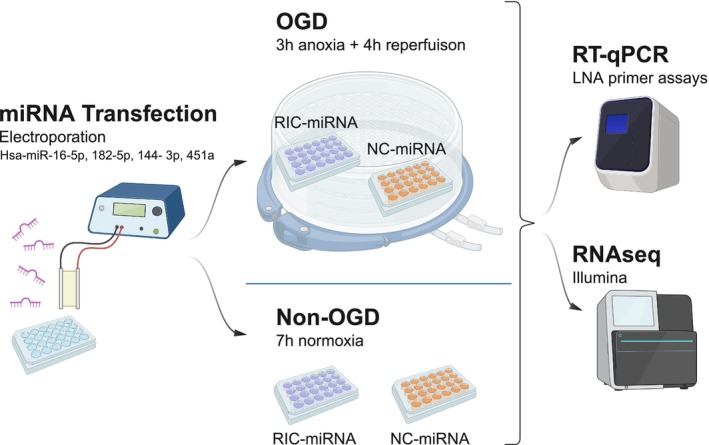
Schematic overview of the full study design. HBMECs were transfected by electroporation, then exposed to oxygen–glucose deprivation (OGD, 3 h anoxic incubation followed by 4 h normoxia) or non‐OGD (7 h normoxia). Firstly, RT‐qPCR was conducted to confirm miRNA transfection, then RNA sequencing was conducted to assess differences between RIC‐miRNA transfected vs. NC‐miRNA transfected and OGD vs. non‐OGD treated HBMECs. Figure created with biorender.com.

The effect of RIC upregulated miRNAs (RIC‐miRNAs) was studied in an in vitro stroke model consisting of primary HBMECs exposed to oxygen–glucose deprivation (3 h) followed by reperfusion (4 h) (OGD) mimicking ischemic stroke and reperfusion. This OGD protocol (3 h anoxia, 4 h reperfusion) was selected to achieve a balance between reducing cell viability and maintaining adequate cell survival for downstream RNA sequencing. Prior to OGD, the HBMECs were transfected with either a cocktail of RIC upregulated miRNAs (miR‐16‐5p, miR‐144‐3p, miR‐182‐5p, and miR‐451a (RIC‐miRNA)) or a scrambled negative control miRNA (NC‐miRNA, Qiagen). RIC‐miRNAs or NC‐miRNA transfected HBMECs cultured under normoxic conditions for 7 h (non‐OGD) were used as control conditions (Figure 1). Then, total RNA was extracted for miRNA transfection efficiency quantification (RT‐qPCR) and differential mRNA expression changes using RNA sequencing (RNA‐seq).

### 2.2 Cell Culture and Handling

HBMECs derived from the cerebral cortex of a healthy 38‐year‐old Caucasian male donor (HIV‐1, HBV, HCV, and mycoplasma negative; cAP‐0002, Angio‐Proteomie, Boston, MA, USA) were used in passage 7. HBMECs were maintained in complete Microvascular Endothelial Cell Growth Medium (EGM, Cellovations cells and media—PELOBiotech—Cat.: PB‐MH‐100‐4099, Planegg, Germany) at 37°C, 5% CO_2_, and 95% humidity. Before seeding, all flasks and plates were coated in 2 μg/cm^2^ Fibronectin Bovine Protein, Plasma (Cat.: 33010018, Gibco, Billings, MT, USA). For testing, HBMECs were detached using Accutase (Sigma‐Aldrich St. Louis, MO, USA), spun at 800 *g* for 5 min, and resuspended in 1 mL Mg^2+^‐ and Ca^2+^‐free dPBS (dPBS, Alfa Aesar, Thermo Fisher Scientific, Ward Hill, MA, USA) before electroporation and seeding in appropriate plates.

### 2.3 Transfection and Oxygen–Glucose Deprivation

At passage 7, 80%–90% confluent HBMECs were transfected by electroporation using a BTX 830 square wave electroporator (45‐0052INT, BTX, Holliston, MA, USA). All miRCURY LNA miRNA mimics were acquired from Qiagen (Hilden, Nordrhein‐Westfalen, Germany) and were used in an absolute concentration of 20 nM for all transfected conditions. Electroporation was carried out as follows: After splitting, 8 × 10^5^ cells were resuspended in dPBS (final volume 800 μL) and mixed with a cocktail (RIC‐miRNAs) of hsa‐miR‐16‐5p (YM00472578‐ADA), hsa‐miR‐144‐3p (YM00473082‐ADA), hsa‐miR‐182‐5p (YM00470840‐ADA), and hsa‐miR‐451a (YM00471387‐ADA) or Negative Control 4 (YM00479903‐ADA, Negative control, NC‐miRNA). The suspended cells were transferred to 4 mm electroporation cuvettes (45–0126, BTX), electroporated with 1 square wave pulse of 80 V for 20 ms and seeded 1 × 10^5^ cells/well in a 24 well plate (Clear, Sarstedt, Nümbrecht, Germany), which already contained 400 μL warm EGM. Two hours after seeding, the media was changed to fresh EGM. For the oxygen–glucose deprivation (OGD) assay, HBMECs were grown for 20 h before media was replaced with Endothelial Cell Basal Medium—Glucose and Phenol Red Free (ECBM, cat#: GPF1168b, Cell Biologics Inc. Chicago, IL, USA). The plates had their lids removed and were placed in a hypoxia chamber (STEMCELL Technologies, Grenoble, France) and flushed for 45 min with anoxic gas (5% CO_2_, 95% N_2_) and then tied off to avoid gas exchange. The oxygen level was monitored with Anaerotest Strips (Millipore, Burlington, MA, USA). HBMECs were incubated at anoxic conditions for 3 h in total. Then, the plates were moved to normoxic conditions and incubated for 4 h, mimicking the reperfusion phase. A normoxia (non‐OGD) plate was run as a control for the experiments for 7 h in the CO_2_‐incubator with both RIC‐miRNA and NC‐miRNA transfected HBMECs.

### 2.4 miRNA Quantification

RT‐qPCR was used to estimate the efficiency of transfection by electroporation. After electroporation with RIC‐miRNAs or NC‐miRNA combined with OGD or non‐OGD treatment, RNA was extracted using Total RNA purification kit (Norgen Biotek Corp., Thorold, ON, Canada). The cells were immediately lysed in RL Lysis buffer (Norgen Total RNA purification kit) and the cell lysate was stored at −80°C prior to RNA purification. Purification was done according to the manufacturer's protocol, including an additional wash step and additional DNase I treatment (RNase‐Free DNase I Kit, Product #25710, Norgen). For each RT‐qPCR reaction, 20 pM spike‐ins: ath‐miR159a and osa‐miR414 (MIMAT0000177 and MIMAT0001330, Integrated DNA Technologies, Coralville, IA, USA) were added after purification to avoid contamination of samples, which were used for RNA‐seq. A total of 100 ng purified RNA of each condition (RIC‐miRNA transfected and NC‐miRNA transfected) was used as the input for Reverse Transcription (RT) reaction (miRCURY LNA RT Kit, Qiagen). RT reaction was run according to the manufacturer's protocol. The qPCR was run with miRCURY LNA miRNA PCR assays (Qiagen) for the RIC‐miRNAs; miR‐16‐5p (YP00205702), miR‐144‐3p (YP00204754), miR‐182‐5p (YP00206070), and miR‐451a (YP02119305), and the spike‐ins: ath‐miR‐159a and osa‐miR‐414 (YP02100546 and YP02104365, respectively). The reactions were run according to the manufacturer's protocol. The qPCR reaction was conducted using the miRCURY LNA SYBR Green Kit (Qiagen) and run on a Mx3005P qPCR instrument (Agilent Technologies, Santa Clara, CA, USA) according to the manufacturer's protocol. Samples were run in technical duplicates of biological triplicates. Fold change expression analysis (2^−ΔΔCt^) was conducted with spike‐ins for normalization of miRNAs expression [30]. The RT‐qPCR data was analyzed in GraphPad prism 9.0 (La Jolla, CA, USA) using ordinary two‐way ANOVA with Tukey's multiple comparison test.

### 2.5 RNA Sequencing

After electroporation and OGD/non‐OGD, RNA was extracted from the HBMECs as described above and stored in elution tubes (Norgen Biotek Corp.) at −80°C until sequencing was performed. Library preparation and RNA‐seq were performed by the NGS Core Center, Department of Molecular Medicine, Aarhus University Hospital. Briefly, 10 μL of 24–36 ng/μL RNA was provided, normalized and used as input for library preparation using the Illumina Stranded Total RNA Prep with Ribo‐Zero Plus. The libraries were paired‐end sequenced on a Novaseq S2 flowcell (50 million read‐pairs, 150 bp). The resulting FASTQ raw reads were qualified and adapter trimmed using Trim‐galore with default settings (https://github.com/FelixKrueger/TrimGalore/). Next, transcript abundance was estimated by quasi‐mapping to the hg38 genome build using Salmon [31]. The transcript counts were loaded into R by Tximeta [32] and the transcript abundances were summarized to gene level. Next, filtering of low‐expression genes was performed (expressed genes: more than 50 gene counts in 3 or more samples). The gene counts of the expressed genes were then used as input for DEseq2 [33] to identify differentially expressed genes (*p*_adj_ < 0.05, log2FC I > 0.5 I). The differentially expressed genes were visualized by volcano plots (https://github.com/kevinblighe/EnhancedVolcano), and the intersection of overlapping DEGs, between the different contrasts, were visualized by Venn diagrams (Venn diagram tool [34]). Pathway and Ontology enrichment analysis were carried out on DEGs using Enrichr [35, 36, 37].

### 2.6 In Silico Target Prediction

Two complementary in silico approaches were employed to identify gene targets of the four selected RIC‐miRNAs (hsa‐miR‐16‐5p, hsa‐miR‐144‐3p, hsa‐miR‐182‐5p, and hsa‐miR‐451a). First, experimentally validated miRNa‐mRNA interactions were retrieved using miRnet (Xia lab, McGill University, Montreal, Quebec, Canada [38]) based on miRTarBase v. 8.0 [39]. Second, putative novel targets were identified using prediction databases TargetScan (v7.2, March 2018) [40, 41] and miRDB [42, 43] and further assessed using the in silico predictor tool miRanda [44]. For miRanda analysis, 3′ untranslated region (3′UTR) sequences of all human transcripts were downloaded from UCSC Genome Browser (hg19), and only targets with a sequence complimentary score (SCS) > 110 and a minimum free energy (FE) < −10 were considered. Predicted targets were compared across all approaches, and genes identified by at least two methods were retained. To ensure relevance to the experimental model, the final list of verified and predicted targets was filtered to include only mRNAs expressed in HBMECs, as determined from HBMEC RNA‐seq data. This combined strategy was applied to balance sensitivity in target identification with increased confidence in predicted miRNA–mRNA interactions.

### 2.7 Weighted Gene Correlation Network Analysis

The Weighted Gene Correlation Network Analysis (WGCNA, version 1.70.3) [45] technique was employed to find co‐expression patterns among genes using the RNA‐seq data as input, and hereafter, to establish connections between these gene modules and the distinct treatments of the HBMECs. Input data consisted of variance‐stabilized counts for all identified genes. The construction of signed co‐expression networks followed a one‐step approach. The determination of adjacency involved selecting an appropriate soft thresholding power to ensure an approximate scale‐free topology. Gene clustering was conducted based on the signed Topology Overlap Matrix through hierarchical clustering, thereby identifying modules utilizing the blockwiseModules function. To quantify module characteristics, module eigengenes were computed using the moduleEigengenes function. The significance of eigengenes and the corresponding *p*‐values were calculated for specific module‐trait relationships. For each targeted module, the intramodular connectivity and gene significance concerning traits were extracted.

## 3 Results

### 3.1 Transfected miRNAs Are Still Present After Oxygen–Glucose Deprivation

**FIGURE 2:**
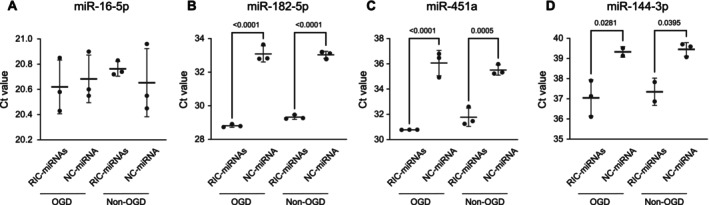
HBMECs are readily transfected with miRNAs using electroporation. Three out of four miRNAs in the RIC‐miRNA cocktail showed a significant increase in HBMECs in normoxia and after OGD. (A) miR‐16‐5p is endogenously highly expressed (low Ct‐values) in HBMECs regardless of transfection status or oxygen tension, thus we saw no expression fold change between the groups. (B–D) miR‐182‐5p, miR‐451a, and miR‐144‐3p were lowly expressed in NC‐miRNA regardless of oxygen tension but increased significantly with transfection with RIC‐miRNAs. Data is presented as mean ± SD.

As RIC elicits a systemic response and simultaneously affects several miRNAs, which might be necessary to convey the protective effect we previously have observed from RIC‐EVs [24], we chose to study a cocktail of the RIC upregulated miRNAs. This cocktail consisted of equal amounts of the four RIC‐miRNAs (miR‐16‐5p, miR‐182‐5p, miR‐144‐3p, and miR‐451a), which were transferred to the cells in vitro via a novel transfection protocol using electroporation. To determine the transfection efficacy in HBMECs after Oxygen–Glucose Deprivation (OGD), the RIC‐miRNA levels were quantified in HBMECs transfected with RIC‐miRNAs or NC‐miRNAs and then subjected to either OGD or left at normal culturing conditions (non‐OGD). As expected, transfection with the NC‐miRNA did not change RIC‐miRNA levels. Interestingly, miR‐16‐5p was endogenously expressed at high levels (Ct value of approx. 20.5) in all samples regardless of transfection treatment or oxygen tension and showed no detectable expression fold change after treatment (2^−Δ∆Ct^ < 0.2 for all comparisons). In contrast, the remaining three RIC‐miRNAs (miR‐182‐5p, miR‐144‐3p, and miR‐451a) were increased in HBMECs after transfection with RIC‐miRNAs, regardless of oxygen tension. For miR‐451a and miR‐182‐5p, the fold‐change increase was in the range of ~11 to ~32 with higher fold changes in the OGD condition (Table 1). The transfection efficacy for miR‐144‐3p was lower compared to the others; however, the Ct values in both RIC‐miRNAs and NC‐miRNA transfected cells remained high at the limit of RT‐qPCR detection (Figure 2 and Table 1, see Table S1 for all 2^−∆∆Ct^ values). In conclusion, all miRNAs, except for miR‐16‐5p, were successfully transfected and remained elevated in the cells throughout the OGD treatment.

**TABLE 1:** Transfection efficiency of RIC‐miRNAs in HBMECs after OGD and non‐OGD. Average Ct values and expression fold change (2^−∆∆Ct^) values for qPCR analysis of OGD/non‐OGD treated RIC‐miRNA and NC‐miRNA transfected HBMECs. Ct values shown as mean of biological triplicates (±SD). Expression fold change is calculated using spike‐in miRNAs for normalization.

miRNA	Ct, RIC‐miRNA transfected	Ct, NC‐miRNA transfected	2^−∆∆Ct^
miR‐16‐5p			
OGD	20.62 (±0.21)	20.68 (±0.19)	< 0.2
Non‐OGD	20.76 (±0.06)	20.65 (±0.27)	< 0.2
miR‐144‐3p			
OGD	37.05 (±0.90)	39.33 (±0.26)	3.3
Non‐OGD	37.35 (±0.68)	39.45 (±0.34)	2.6
miR‐182‐5p			
OGD	28.81 (±0.09)	33.09 (±0.48)	15.6
Non‐OGD	29.32 (±0.13)	33.03 (±0.20)	11.2
miR‐451a			
OGD	30.78 (±0.01)	36.06 (±1.00)	32.2
Non‐OGD	31.77 (±0.73)	35.51 (±0.41)	11.4

### 3.2 RIC‐miRNAs Change the Gene Expression Profiles in OGD Treated HBMECs

**FIGURE 3:**
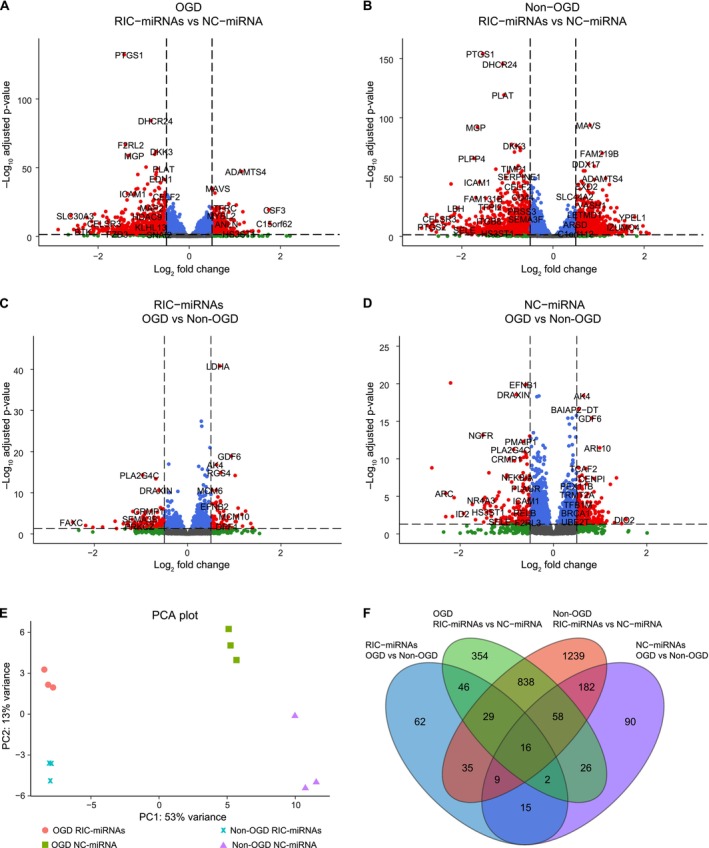
Transfection with RIC‐miRNAs changes gene expression profiles in HBMECs. (A–D) Transfection with RIC‐miRNAs gives rise to more DEGs than oxygen tension (OGD/non‐OGD). Red dots: DEGs defined as a log_2_FoldChange (log_2_FC) < −0.5 and > 0.5 and an adjusted *p*‐value (*p*_adj_) < 0.05. Blue, gray and green dots: Not differentially expressed. Total = 14 516 genes. (E) Principal component analysis (PCA) visualizes the large separation between the groups where the transfection treatment creates a larger variance than oxygen tension. (F) Venn diagram showing common differentially expressed genes (DEGs) in all of the analyses.

Alterations in gene expression in RIC‐miRNA transfected HBMECs after reperfusion following OGD were studied by RNA sequencing (RNA‐seq) of transfected HBMECs. To investigate the changes in gene expression introduced by the different culturing conditions and the miRNA cocktail transfection, we identified differentially expressed genes (DEGs) between the different culturing and transfection conditions (Figure 3A–D). In each comparison, genes with an adjusted *p* < 0.05 (*p*_adj_ < 0.05) and a log_2_FoldChange (log_2_FC) of < −0.5 or > 0.5 were deemed differentially expressed. From this, we observed that OGD treated RIC‐miRNA transfected HBMECs resulted in 1369 DEGs when compared to NC‐miRNA transfected cells, while 2406 DEGs were observed in non‐OGD treated RIC‐miRNA transfected vs. NC‐miRNA transfected HBMECs. In contrast, for the RIC‐miRNA transfected HBMECs, OGD compared to non‐OGD only resulted in 214 DEGs, while in NC‐miRNA transfected HBMECs a total of 398 DEGs were found when comparing OGD and non‐OGD HBMEC mRNA expression.

Principal component analysis (PCA) using all expressed genes of each sample as input, resulted in a clear clustering into the four distinct groups, with most of the variance in the dataset resulting from transfection of RIC‐miRNAs or NC‐miRNA (PC1), whereas the second largest variance arose from OGD or non‐OGD treatment (PC2) (Figure 3E).

Overlapping and unique DEGs for each condition were visualized in a Venn diagram that showed the largest difference in DEGs between RIC‐miRNAs and NC‐miRNA transfected HBMECs for both OGD treated and non‐OGD treated cells (Figure 3F). Many of the DEGs, which were present in the RIC‐miRNAs groups, were shared regardless of oxygen tension (838 DEGs). Noticeable differences between the OGD‐ and non‐OGD treated HBMECs were also observed regardless of transfection status.

### 3.3 RIC‐miRNA Transfection Specifically Changes Gene Expression During OGD

**FIGURE 4:**
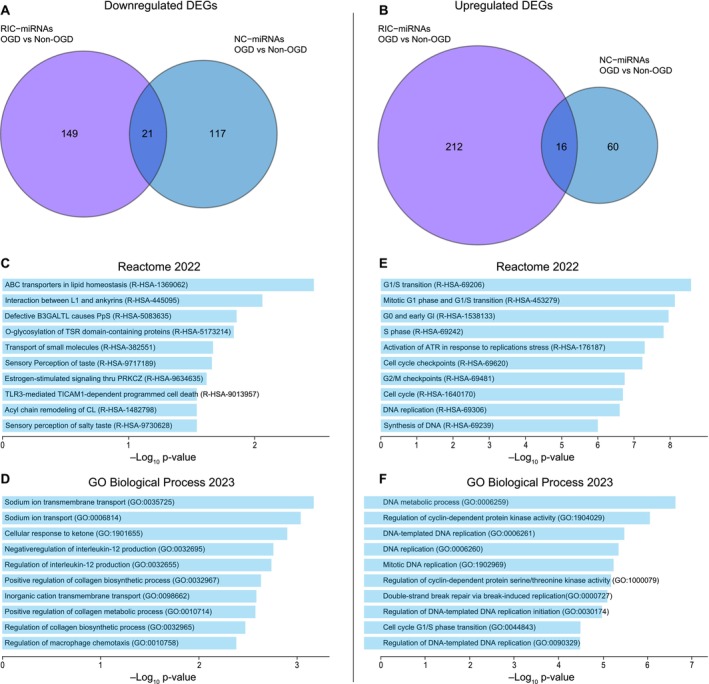
RIC‐miRNAs transfection of HBMECs subjected to OGD changes the expression of genes involved in the cell cycle. (A) Venn diagram showing the difference in downregulated genes between OGD vs. non‐OGD for RIC‐miRNAs vs. NC‐miRNA. (B) Venn diagram showing the difference in upregulated genes between OGD vs. non‐OGD for RIC‐miRNAs versus NC‐miRNA. Reactome 2022 (C) and GO biological process 2023 (D) enrichment analysis of downregulated DEGs in HBMECs after transfection with RIC‐miRNAs and exposure to OGD. Reactome 2022 (E) and GO biological process 2023 (F) enrichment analysis of DEGs in HBMECs after transfection with RIC‐miRNAs and exposure to OGD. HGNC gene symbols were used as input for the enrichment analysis [46].

As OGD mimics the early stages of stroke, the gene response related to this condition, with and without the effect of RIC‐miRNAs, could point to possible early protective pathways of RIC. Comparing DEGs in RIC‐miRNA HBMECs that were specific for the OGD condition with the DEGs after NC‐miRNA transfection showed that out of the 575 DEGs, 287 were downregulated while 288 were upregulated, of which 149 and 212 were specific for the RIC‐miRNA treatment, respectively (Figure 4A,B). For the downregulated DEGs, which include the direct effect for the miRNAs, enrichment analysis (Figure 4C,D) showed several different pathways, of which the sodium (and cation) transport and regulation of interleukin‐12 (IL‐12) were of special interest in regard to stroke and cytoprotection. In contrast, enrichment analyses of the upregulated DEGs (Figure 4E,F) showed that all the top 10 pathways and ontologies were involved in cell cycle regulation and DNA replication, pointing towards these biological pathways as the main effects of the selected four RIC‐miRNAs. These DEGs were all upregulated in RNA‐seq data, meaning that they were most likely derivative effects of the RIC‐miRNAs specifically during OGD treatment and that cell cycle is positively regulated, which may lead to increased cell proliferation and survival. In all, this points towards an early response to the RIC‐miRNAs from the downregulated genes, while the upregulated genes are involved in beneficial pathways with a longer horizon.

### 3.4 Target Prediction Reveal the Direct Effect of the Selected RIC‐miRNAs in HBMECs Subjected to OGD

**FIGURE 5:**
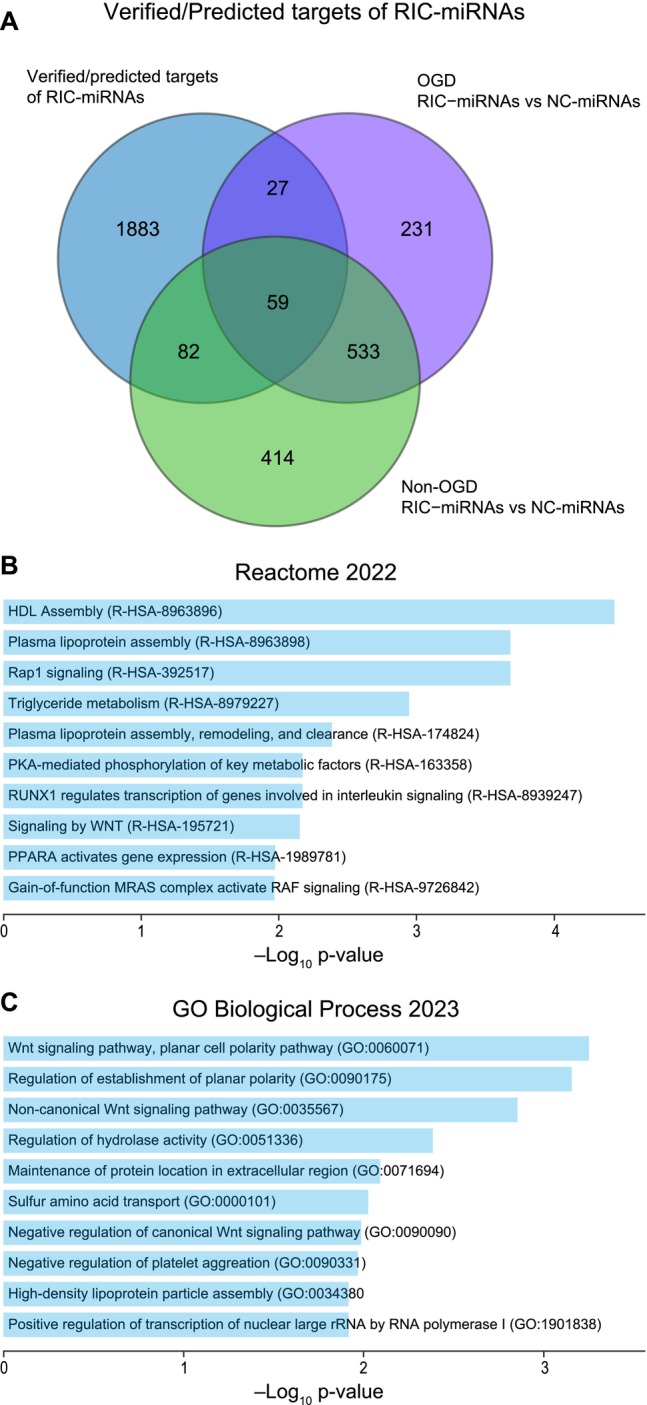
First layer response to RIC‐miRNA in endothelial cells subjected to OGD. (A) Venn diagram showing the overlap between the verified/predicted mRNA targets of the four RIC‐miRNAs and the downregulated DEGs following RIC‐miRNA transfection or NC‐miRNA transfection and OGD or non‐OGD. Reactome 2022 (B) and GO biological process 2023 (C) enrichment analysis of the 27 downregulated DEGs specific for the RIC‐miRNA and OGD condition.

To reveal DE mRNAs directly affected by the transfected RIC‐miRNAs, we first identified mRNA targets of the 4 RIC‐miRNAs using a database of verified miRNA‐mRNA targets (1451 targets). As not all miRNA target sites in mRNAs have been experimentally verified, we also used tools to find predicted targets of the four RIC‐miRNAs. Added together, 2051 verified and/or predicted targets of the RIC‐miRNAs were found and compared with the DEGs in the RNA‐seq data. To get the primary effects of the miRNAs, only mRNA targets which were downregulated (log_2_FC < −0.5) were included in the analysis resulting in 168 downregulated genes after RIC‐miRNA transfection of HBMECs when disregarding culturing conditions. Of these, 27 genes were specifically downregulated after OGD in RIC‐miRNA transfected HBMECs, and thus can be interpreted as the specific response to RIC‐miRNAs during OGD (Figure 5A). These genes can be considered the first layer response to RIC‐miRNAs with implications in diverse pathways (Figure 5B,C) of which the most interesting involves the Wnt signaling pathway, which is crucial for cell fate as well as cell planar polarity involved in development and cell migration.

### 3.5 Several Gene Modules Are Strongly Correlated With RIC‐miRNAs During OGD

**FIGURE 6:**
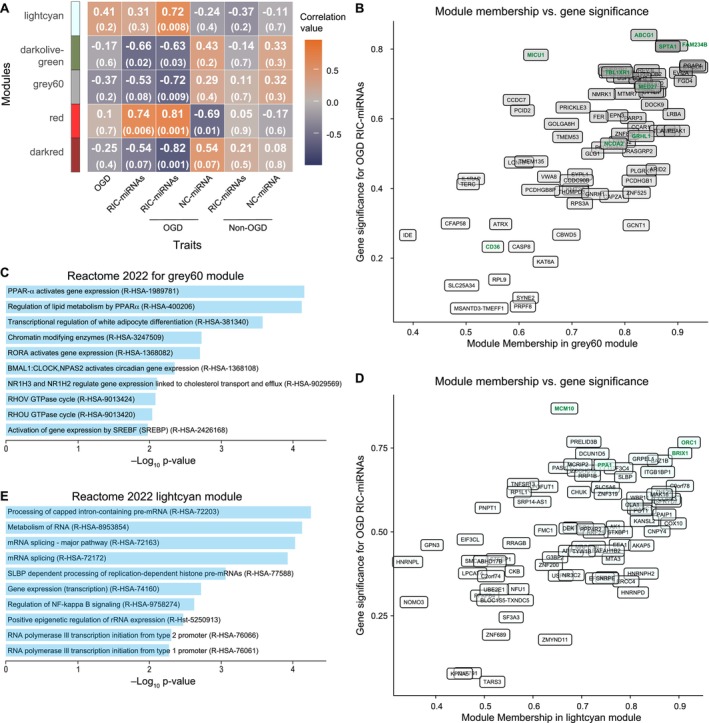
Weighted gene correlated network analysis of DEGs in RIC‐miRNA transfected HBMECs. (A) Gene modules, which were significantly up‐ or downregulated, in relation to OGD treatment and RIC‐miRNA transfection. Only grey60 and lightcyan were chosen for further analysis, as they were the only modules that were significantly regulated when taking both RIC‐miRNA transfection and OGD into account. (B) Module membership for the gene module grey60 showing the included genes and their significance for OGD RIC‐miRNA DEGs. DEGs of special interest are highlighted in green. (C) Reactome 2022 Enrichment analysis for grey60 showed that the PPAR‐α pathway, among others, was significantly affected. (D) Module membership analysis for lightcyan, showing that the PPA1 gene both had a high module‐membership and high gene significance for anoxia. DEGs of special interest are highlighted in green. (E) Reactome 2022 enrichment analysis of lightcyan DEGs showed their involvement in regulation of mRNA splicing mechanisms.

To further study the impact of the RIC‐miRNAs, weighted gene correlation network analysis (WGCNA) was carried out to identify intricate co‐expressed groups of genes. The gene expression data was used as input and co‐expressed genes, termed modules, were then correlated to treatment (OGD or non‐OGD), transfection (RIC‐miRNAs or NC‐miRNA), or a combination (OGD RIC‐miRNAs, OGD NC‐miRNA, non‐OGD RIC‐miRNAs, non‐OGD NC‐miRNA) to identify if these conditions could be linked to specific gene expression patterns (Figure S1 for full WGCNA data). We focused mainly on modules that correlated with OGD RIC‐miRNAs. Three modules were negatively correlated to OGD RIC‐miRNAs (darkolivegreen, grey60 and darkred) while two modules were positively correlated (lightcyan and red) (Figure 6A). The genes within each module were then used as input for enrichment analysis. Interestingly, the grey60 module (Figure 6B), consisting of genes mainly downregulated in the OGD RIC‐miRNAs condition, was significantly enriched in pathways related to lipid metabolism, more specifically the Peroxisome proliferator‐activated receptor alpha (PPAR‐α) pathway (Figure 6C). Furthermore, the lightcyan module (Figure 6D), consisting of genes mainly upregulated in OGD RIC‐miRNAs, was enriched in mRNA splicing processes (Figure 6E). Examining gene module membership and their association with the OGD treated RIC‐miRNAs transfected HBMECs, several genes were identified that exhibited significant relevance to both factors, underscoring their importance. Notably, within the grey60 module, genes such as SPTA1, FAM234B, ABCG1 and MICU1 were found, all linked to cellular membrane organization [47], lipid/cholesterol metabolism [48], and mitochondrial calcium turnover [49]. Similarly, the lightcyan module contained genes like MCM10, ORC1 and BRIX1, known for functions in DNA repair/replication [50], cell cycle progression and ribosome biogenesis [51, 52]. Interestingly, the gene PPA1, an inorganic pyrophosphatase, also stood out, potentially providing an alternate energy source during an energy‐deprived state like AIS [53]. This module‐based expression pattern, with grey60 genes downregulated and lightcyan genes upregulated, might facilitate cellular recovery and adaptation. Collectively, these mechanisms could play a role in safeguarding endothelial cell function during ischemia–reperfusion, as further detailed in the discussion.

## 4 Discussion

Based on previous studies of the cytoprotective effects of RIC, we hypothesized that specific RIC‐induced circulating miRNAs may alter the transcriptome of HBMECs to convey cytoprotective effects to withstand damaging ischemia and reperfusion.

To observe the primary effects of the RIC‐miRNAs during OGD, the downregulated DEGs were identified. Here, downregulation of SLC24A1, SCNN1D, and SCN9A that are involved in cation transport into the cell, including sodium transport, could protect HBMECs from the detrimental effects of increased intracellular sodium [54]. Furthermore, the downregulated DEGs included the genes encoding CMKLR1, PIBF1, and TLR3, which are involved in regulation of IL‐12, a pro‐inflammatory cytokine. As IL‐12 is thought to exacerbate the degradation of brain cells during the acute phase of stroke, attenuation of this cytokine could have protective effects during stroke [55].

Transfection with RIC‐miRNA and subsequent OGD treatment also led to many upregulated DEGs. These are primarily involved in cell cycle activation. Especially positive regulators of the cell cycle were upregulated after OGD, which implies re‐activation of the pathways. Several genes involved in the G1/S transition were upregulated, including CDC25A, CCNE1, and CCNE2. Under anoxic conditions, hypoxia‐inducible factor downregulates cell cycle pathways to conserve energy (reviewed by Druker et al. [56]). Our data point towards an activation of the cell cycle specifically tied to RIC‐miRNA transfection of HBMECs after OGD.

The impact of the RIC‐miRNAs was further investigated using WGCNA that identified two gene networks (grey60 and lightcyan modules) specifically changed during OGD. Enrichment analysis of the genes in the grey60 module, which consists mainly of downregulated genes, identified pathways related to lipid metabolism, particularly the PPAR‐α pathway. The collective downregulation of PPAR‐α pathway genes in this module like NCOA2, TBL1XR1, CD36, GRHL1, and MED27 during OGD in RIC‐miRNA transfected HBMECs suggests a complex adaptive response possibly involving reduction of energy demand in HBMECs. On the other hand, we also observed a module, lightcyan, that was positively associated with OGD, and thus, a general upregulation of the genes within this module was observed. The genes in this module were enriched in DNA repair, cell cycle progression and ribosome biogenesis, suggesting an adaptive shift towards pathways facilitating cellular maintenance and proliferation possibly to ensure functionality after OGD. Notably, the PPA1 gene is interesting in this adaptive response as it belongs to the family of inorganic pyrophosphatases that plays an important role in energy metabolism [53, 57]. Thus, we speculate that the downregulation of genes in the grey60 module and upregulation of genes in the lightcyan module could contribute to alternative cellular energy adaptation and recovery during and following OGD.

The current study comes with some limitations, the first being the low translational value as we are only studying a simplified in vitro stroke model in a monoculture of endothelial cells. Moreover, the use of primary cells derived from a single 38‐year‐old Caucasian male donor limits the generalizability of the findings, particularly with respect to age‐, sex‐, and ethnicity‐dependent biological variability. On the other hand, we are investigating the transcriptomic changes in primary human brain cells, which ensures a relevant genetic background that increases the translational value of the study compared to animal models of stroke. In addition, the use of cell models for miRNA effect screening and hypothesis generation reduces the ethical concern of animal experimentation. Although a more complex system (e.g., animal stroke model, BBB cell culture model) would include network responses, the use of this simplified system for miRNA effect screening ensures that we obtain knowledge of the endothelial response to the RIC‐miRNAs during OGD. This is important as HBMECs play a critical role in stroke pathophysiology and would be the first cells to be affected by systemically applied stroke treatment. The observed transcriptomic changes cannot be ascribed to a single RIC‐miRNA, as we only studied the effect of the cocktail of RIC‐miRNAs. It is also uncertain if we are looking at a combined effect due to all or several of the RIC‐miRNAs. However, no changes in miR‐16‐5p were observed and miR‐144‐3p expression was generally low in all samples, making the quantification of this miRNA questionable. Single transfections could provide data on the single effects of the miRNAs, but maybe not the full picture, as we speculate that the protective effect, previously observed in RIC‐EVs, can be a synergistic effect of several DE miRNAs. Accordingly, we suggest that these changes are not isolated events, but simultaneous activation may be the key to understanding the cytoprotection of RIC‐EVs. While our analyses are based solely on RNA sequencing and may not directly reflect corresponding changes in protein expression or activity, the identified transcriptomic patterns suggest that both positive regulation of the cell cycle and the utilization of alternative energy sources may be beneficial for endothelial cell survival during OGD.

In conclusion, we found substantial changes in the gene expression profile in HBMECs transfected with RIC‐miRNAs, as well as during in vitro modeling of ischemia and reperfusion. These changes were linked to positive cell cycle regulation and a possible switch to alternative energy generating pathways that may help HBMECs cope with the physiological stress during stroke. This study can serve as a basis for investigating novel avenues of cell cycle regulation and alternative energy metabolism for stroke treatments.

## 5 Perspectives

The molecular changes caused by the introduced RIC‐miRNAs during OGD point towards interesting pathways that could explain the beneficial effects of RIC reported in both experimental stroke models and clinical trials. Especially, the regulation of genes involved in cell cycle activation and in pathways of alternative energy metabolism is interesting as these pathways could in part underlie the beneficial effects of RIC. The current study suggests a potential role for miRNAs in stroke treatment and suggests new studies of cytoprotection in stroke that might be most effective when activated simultaneously.

## Funding

This work was supported by the Sino‐Danish Center for Education and Research, and The Novo Nordisk Foundation, Denmark (grant NNF 15OC0016674).

## Supporting information


**Figure S1:** The full weighted gene correlated network analysis (WGCNA) of DEGs in RIC‐miRNA transfected HBMECs.

**Table S1:** Transfection efficacy (Δ*Δ*Ct) of transfected HBMECs, which were exposed to OGD or non‐OGD.

## Data Availability

The data supporting the findings of this study are available from the corresponding author upon reasonable request for research purposes. The authors have no conflicts of interest to disclose.
