# Supplementary material for: Insights Into Remote Ischemic Conditioning miRNA Effects on Brain Endothelial Cells During Ischemia–Reperfusion
Source: Microcirculation. 2026 May 1;33:e70060. doi: 10.1111/micc.70060 (PMC13135127; doi:10.1111/micc.70060)
Supplement: Supplementary file 1 — Figure S1: The full weighted gene correlated network analysis (WGCNA) of DEGs in RIC‐miRNA transfected HBMECs. [file MICC-33-e70060-s001.docx]

grey thistle1 darkorange

yellow cyan lightcyan purple lightyellow

pink bisque4 steelblue sienna3 ivory plum2

lightsteelblue1

brown4 lightcyan1 darkorange2 darkslateblue

turquoise darkolivegreen

grey60

tan darkgrey

salmon

plum1 blue paleturquoise

Modules

white lightgreen

brown midnightblue

magenta

red violet skyblue floralwhite darkmagenta orangered4

green mediumpurple3

orange navajowhite2 yellowgreen saddlebrown

darkred thistle2 skyblue3 salmon4 darkgreen palevioletred3 greenyellow darkturquoise

royalblue

black

Correlation value

0.5


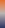


0.0

-0.5

| **0**  **(1)**  **0.41**  **(0.2)**  **0.3**  **(0.3)**  **0.95 (3e-06) 0.83 (8e-04) 0.41**  **(0.2)**  **0.08**  **(0.8)**  **0.49**  **(0.1)**  **0.38**  **(0.2)**  **0.42**  **(0.2)**  **0.48**  **(0.1)**  **0.53**  **(0.08)**  **0.68**  **(0.02)**  **0.46**  **(0.1)**  **-0.14**  **(0.7)**  **0.55**  **(0.06)**  **-0.18**  **(0.6)**  **-0.25**  **(0.4)**  **0.25**  **(0.4)**  **-0.13**  **(0.7)**  **-0.17**  **(0.6)**  **-0.37**  **(0.2)**  **-0.25**  **(0.4)**  **-0.26**  **(0.4)**  **-0.18**  **(0.6)**  **0.16**  **(0.6)**  **0.26**  **(0.4)**  **0.04**  **(0.9)**  **0.23**  **(0.5)**  **-0.46**  **(0.1)**  **-0.71**  **(0.01)**  **-0.26**  **(0.4)**  **-0.55**  **(0.06)**  **0.1**  **(0.7)** | **-0.16 -0.22**  **(0.6) (0.5)**  **0.22 0.26**  **(0.5) (0.4)**  **-0.07 0.28**  **(0.8) (0.4)**  **0.01 0.68**  **(1) (0.01)**  **0.12 0.39**  **(0.7) (0.2)**  **0.31 0.72**  **(0.3) (0.008)**  **-0.39 0.29**  **(0.2) (0.4)**  **-0.36 0.46**  **(0.3) (0.1)**  **-0.62 0.15**  **(0.03) (0.6)**  **-0.3 0.08**  **(0.3) (0.8)**  **-0.34 0.22**  **(0.3) (0.5)**  **-0.45 -0.21**  **(0.1) (0.5)**  **-0.26 0.16**  **(0.4) (0.6)**  **-0.63 -0.04**  **(0.03) (0.9)**  **-0.36 -0.17**  **(0.2) (0.6)**  **-0.02 0.44**  **(1) (0.2)**  **-0.16 -0.14**  **(0.6) (0.7)**  **-0.18 -0.14**  **(0.6) (0.7)**  **-0.19 -0.07**  **(0.6) (0.8)**  **-0.98 -0.57**  **(9e-09) (0.05)**  **-0.66 -0.63**  **(0.02) (0.03)**  **-0.53 -0.72**  **(0.08) (0.009)**  **0.57 -0.06**  **(0.05) (0.8)**  **0.59 0.02**  **(0.04) (0.9)**  **0.55 -0.13**  **(0.07) (0.7)**  **0.58 0.28**  **(0.05) (0.4)**  **0.95 0.6**  **(2e-06) (0.04)**  **0.57 0.38**  **(0.05) (0.2)**  **0.32 0.33**  **(0.3) (0.3)**  **0.33 0.32**  **(0.3) (0.3)**  **-0.3 -0.24**  **(0.3) (0.5)**  **0.39 0.38**  **(0.2) (0.2)**  **0.68 0.27**  **(0.02) (0.4)**  **0.74 0.81**  **(0.006) (0.001)** | | **0.23**  **(0.5)**  **0.21**  **(0.5)**  **0.07**  **(0.8)**  **0.41**  **(0.2)**  **0.57**  **(0.05)**  **-0.24**  **(0.4)**  **-0.2**  **(0.5)**  **0.1**  **(0.7)**  **0.29**  **(0.4)**  **0.4**  **(0.2)**  **0.33**  **(0.3)**  **0.82**  **(0.001)**  **0.62**  **(0.03)**  **0.56**  **(0.06)**  **0.01**  **(1)**  **0.2**  **(0.5)**  **-0.07**  **(0.8)**  **-0.15**  **(0.7)**  **0.36**  **(0.2)**  **0.42**  **(0.2)**  **0.43**  **(0.2)**  **0.29**  **(0.4)**  **-0.22**  **(0.5)**  **-0.32**  **(0.3)**  **-0.08**  **(0.8)**  **-0.09**  **(0.8)**  **-0.3**  **(0.3)**  **-0.34**  **(0.3)**  **-0.06**  **(0.9)**  **-0.85**  **(5e-04)**  **-0.58**  **(0.05)**  **-0.68**  **(0.02)**  **-0.9**  **(6e-05)**  **-0.69**  **(0.01)** | **0.03 -0.04**  **(0.9) (0.9)**  **-0.01 -0.46**  **(1) (0.1)**  **-0.36 0.01**  **(0.3) (1)**  **-0.67 -0.42**  **(0.02) (0.2)**  **-0.25 -0.71**  **(0.4) (0.01)**  **-0.37 -0.11**  **(0.2) (0.7)**  **-0.74 0.65**  **(0.006) (0.02)**  **-0.88 0.31**  **(2e-04) (0.3)**  **-0.87 0.43**  **(2e-04) (0.2)**  **-0.42 -0.06**  **(0.2) (0.9)**  **-0.62 0.06**  **(0.03) (0.9)**  **-0.31 -0.3**  **(0.3) (0.3)**  **-0.47 -0.31**  **(0.1) (0.3)**  **-0.69 0.16**  **(0.01) (0.6)**  **-0.25 0.41**  **(0.4) (0.2)**  **-0.46 -0.18**  **(0.1) (0.6)**  **-0.05 0.25**  **(0.9) (0.4)**  **-0.07 0.36**  **(0.8) (0.3)**  **-0.15 -0.14**  **(0.6) (0.7)**  **-0.56 0.71**  **(0.06) (0.009)**  **-0.14 0.33**  **(0.7) (0.3)**  **0.11 0.32**  **(0.7) (0.3)**  **0.72 -0.44**  **(0.008) (0.2)**  **0.66 -0.36**  **(0.02) (0.2)**  **0.76 -0.55**  **(0.004) (0.06)**  **0.39 -0.58**  **(0.2) (0.05)**  **0.5 -0.79**  **(0.1) (0.002)**  **0.28 -0.32**  **(0.4) (0.3)**  **0.04 -0.31**  **(0.9) (0.3)**  **0.07 0.46**  **(0.8) (0.1)**  **-0.11 0.93**  **(0.7) (1e-05)**  **0.07 0.23**  **(0.8) (0.5)**  **0.52 0.12**  **(0.08) (0.7)**  **0.05 -0.17**  **(0.9) (0.6)** | |
| --- | --- | --- | --- | --- | --- |
| **-0.53** | **0.38** | **-0.18** | **-0.42** | **0.62** | **-0.02** |
| **(0.08)** | **(0.2)** | **(0.6)** | **(0.2)** | **(0.03)** | **(1)** |
| **-0.43** | **0.28** | **-0.12** | **-0.38** | **0.44** | **0.06** |
| **(0.2)** | **(0.4)** | **(0.7)** | **(0.2)** | **(0.2)** | **(0.9)** |
| **-0.5** | **0.16** | **-0.32** | **-0.27** | **0.51** | **0.08** |
| **(0.09)** | **(0.6)** | **(0.3)** | **(0.4)** | **(0.09)** | **(0.8)** |
| **-0.6** | **0.5** | **0** | **-0.69** | **0.58** | **0.12** |
| **(0.04)** | **(0.1)** | **(1)** | **(0.01)** | **(0.05)** | **(0.7)** |
| **-0.66** | **0.5** | **-0.06** | **-0.71** | **0.64** | **0.13** |
| **(0.02)** | **(0.09)** | **(0.9)** | **(0.01)** | **(0.03)** | **(0.7)** |
| **-0.96** | **0.17** | **-0.52** | **-0.59** | **0.71** | **0.4** |
| **(6e-07)** | **(0.6)** | **(0.09)** | **(0.04)** | **(0.01)** | **(0.2)** |
| **-0.19** | **0.01** | **0.12** | **-0.34** | **-0.12** | **0.33** |
| **(0.6)** | **(1)** | **(0.7)** | **(0.3)** | **(0.7)** | **(0.3)** |
| **-0.17** | **0.11** | **-0.39** | **0.19** | **0.51** | **-0.31** |
| **(0.6)** | **(0.7)** | **(0.2)** | **(0.6)** | **(0.09)** | **(0.3)** |
| **-0.17** | **0.25** | **-0.22** | **0.03** | **0.52** | **-0.32** |
| **(0.6)** | **(0.4)** | **(0.5)** | **(0.9)** | **(0.09)** | **(0.3)** |
| **-0.53** | **0.08** | **-0.35** | **-0.26** | **0.44** | **0.16** |
| **(0.08)** | **(0.8)** | **(0.3)** | **(0.4)** | **(0.1)** | **(0.6)** |
| **-0.4** | **0.01** | **-0.66** | **0.21** | **0.67** | **-0.21** |
| **(0.2)** | **(1)** | **(0.02)** | **(0.5)** | **(0.02)** | **(0.5)** |
| **-0.25** | **-0.54** | **-0.82** | **0.54** | **0.21** | **0.08** |
| **(0.4)** | **(0.07)** | **(0.001)** | **(0.07)** | **(0.5)** | **(0.8)** |
| **-0.42** | **-0.04** | **-0.44** | **-0.04** | **0.4** | **0.08** |
| **(0.2)** | **(0.9)** | **(0.1)** | **(0.9)** | **(0.2)** | **(0.8)** |
| **-0.25** | **-0.36** | **-0.59** | **0.3** | **0.18** | **0.11** |
| **(0.4)** | **(0.3)** | **(0.04)** | **(0.3)** | **(0.6)** | **(0.7)** |
| **0.41** | **-0.12** | **-0.2** | **0.67** | **0.05** | **-0.52** |
| **(0.2)** | **(0.7)** | **(0.5)** | **(0.02)** | **(0.9)** | **(0.08)** |
| **0.42** | **0.31** | **0.09** | **0.4** | **0.27** | **-0.75** |
| **(0.2)** | **(0.3)** | **(0.8)** | **(0.2)** | **(0.4)** | **(0.005)** |
| **0.44** | **0.15** | **-0.1** | **0.6** | **0.27** | **-0.78** |
| **(0.2)** | **(0.6)** | **(0.8)** | **(0.04)** | **(0.4)** | **(0.003)** |
| **-0.62** | **-0.32** | **-0.64** | **-0.07** | **0.28** | **0.43** |
| **(0.03)** | **(0.3)** | **(0.02)** | **(0.8)** | **(0.4)** | **(0.2)** |
| **-0.38** | **-0.04** | **-0.52** | **0.08** | **0.47** | **-0.03** |
| **(0.2)** | **(0.9)** | **(0.08)** | **(0.8)** | **(0.1)** | **(0.9)** |
| **0.25** | **0.31** | **0.06** | **0.22** | **0.3** | **-0.58** |
| **(0.4)** | **(0.3)** | **(0.8)** | **(0.5)** | **(0.3)** | **(0.05)** |
| **-0.31** | **0.52** | **-0.17** | **-0.19** | **0.77** | **-0.41** |
| **(0.3)** | **(0.08) (0.6) (0.5)** | | | **(0.003) (0.2)** | |

OGD Non-OGD

Traits

**Supplementary Figure 6.1**: The full weighted gene correlated network analysis (WGCNA) of DEGs in RIC-miRNA transfected HBMECs.
