# Supplementary material for: Insights Into Remote Ischemic Conditioning miRNA Effects on Brain Endothelial Cells During Ischemia–Reperfusion
Source: Microcirculation. 2026 May 1;33:e70060. doi: 10.1111/micc.70060 (PMC13135127; doi:10.1111/micc.70060)
Supplement: Supplementary file 2 — Table S1: Transfection efficacy (ΔΔCt) of transfected HBMECs, which were exposed to OGD or non‐OGD. [file MICC-33-e70060-s002.docx]

**Supplementary Table 1.1**: Transfection efficacy (ΔΔCt) of transfected HBMECs, which were exposed to OGD or non-OGD.

| **miRNA** | **Transfection** | **Treatment** | **ΔΔCt** |
| --- | --- | --- | --- |
| miR-16-5p | RIC-miRNA vs. NC-miRNA | OGD | -0.11 |
|  | RIC-miRNA vs. NC-miRNA | Non-OGD | -0.14 |
|  | RIC-miRNA | OGD vs. Non-OGD | 0.11 |
|  | NC-miRNA | OGD vs. Non-OGD | 0.07 |
| miR-182-5p | RIC-miRNA vs. NC-miRNA | OGD | 15.55 |
|  | RIC-miRNA vs. NC-miRNA | Non-OGD | 11.18 |
|  | RIC-miRNA | OGD vs. Non-OGD | 0.43 |
|  | NC-miRNA | OGD vs. Non-OGD | 0.05 |
| miR-144-3p | RIC-miRNA vs. NC-miRNA | OGD | 3.29 |
|  | RIC-miRNA vs. NC-miRNA | Non-OGD | 2.59 |
|  | RIC-miRNA | OGD vs. Non-OGD | 0.30 |
|  | NC-miRNA | OGD vs. Non-OGD | 0.09 |
| miR-451a | RIC-miRNA vs. NC-miRNA | OGD | 32.24 |
|  | RIC-miRNA vs. NC-miRNA | Non-OGD | 11.44 |
|  | RIC-miRNA | OGD vs. Non-OGD | 1.00 |
|  | NC-miRNA | OGD vs. Non-OGD | -0.25 |
